# Multiple Indices of Diffusion Identifies White Matter Damage in Mild Cognitive Impairment and Alzheimer’s Disease

**DOI:** 10.1371/journal.pone.0021745

**Published:** 2011-06-30

**Authors:** Laurence O'Dwyer, Franck Lamberton, Arun L. W. Bokde, Michael Ewers, Yetunde O. Faluyi, Colby Tanner, Bernard Mazoyer, Des O'Neill, Máiréad Bartley, D. Rónán Collins, Tara Coughlan, David Prvulovic, Harald Hampel

**Affiliations:** 1 Department of Psychiatry, Psychosomatic Medicine and Psychotherapy, Goethe University, Frankfurt, Germany; 2 Centre for Imaging-Neurosciences and Applications to Pathologies, UMR 6232, CNRS, CEA, University of Caen and Paris Descartes, Paris, France; 3 Cognitive Systems Group, Discipline of Psychiatry, School of Medicine and Trinity College Institute of Neuroscience (TCIN), Lloyd Building, Trinity College Dublin, Dublin, Ireland; 4 Department of Radiology, VA Medical Center, University of California San Francisco, San Francisco, California, United States of America; 5 Liaison Psychiatry Service, Addenbrooke's Hospital, Cambridge, United Kingdom; 6 Cambridgeshire and Peterborough NHS Foundation Trust, Cambridgeshire, United Kingdom; 7 Department of Zoology, Trinity College Dublin, Dublin, Ireland; 8 Centre Hospitalier Universitaire, Caen, France; 9 Institut Universitaire de France, Paris, France; 10 Department of Medical Gerontology, Trinity College Dublin, Dublin, Ireland; Nathan Kline Institute and New York University School of Medicine, United States of America

## Abstract

The study of multiple indices of diffusion, including axial (DA), radial (DR) and mean diffusion (MD), as well as fractional anisotropy (FA), enables WM damage in Alzheimer's disease (AD) to be assessed in detail. Here, tract-based spatial statistics (TBSS) were performed on scans of 40 healthy elders, 19 non-amnestic MCI (MCIna) subjects, 14 amnestic MCI (MCIa) subjects and 9 AD patients. Significantly higher DA was found in MCIna subjects compared to healthy elders in the right posterior cingulum/precuneus. Significantly higher DA was also found in MCIa subjects compared to healthy elders in the left prefrontal cortex, particularly in the forceps minor and uncinate fasciculus. In the MCIa versus MCIna comparison, significantly higher DA was found in large areas of the left prefrontal cortex. For AD patients, the overlap of FA and DR changes and the overlap of FA and MD changes were seen in temporal, parietal and frontal lobes, as well as the corpus callosum and fornix. Analysis of differences between the AD versus MCIna, and AD versus MCIa contrasts, highlighted regions that are increasingly compromised in more severe disease stages. Microstructural damage independent of gross tissue loss was widespread in later disease stages. Our findings suggest a scheme where WM damage begins in the core memory network of the temporal lobe, cingulum and prefrontal regions, and spreads beyond these regions in later stages. DA and MD indices were most sensitive at detecting early changes in MCIa.

## Introduction

Alzheimer's disease (AD) is thought to begin with biochemical and structural changes at the synaptic level which impinge on cognitive function and lead to neuronal death and the degeneration of white matter (WM) tracts [1]. While AD entails significant memory loss, an intermediate state between healthy aging and AD, termed mild cognitive impairment (MCI) has become widely recognized. A significant proportion of those with MCI may represent a prodromal state of AD, with an estimated 10–15% of MCI subjects progressing to dementia every year [2]. Within this framework, an ability to accurately classify MCI subjects into those with and without underlying AD pathology is vital as disease-modifying compounds which target amyloid beta (Aβ) accumulation may only work in the earliest preclinical stages of AD [3].

WM changes may be a key indicator of early pathology [4] and WM damage in AD has been highlighted both in postmortem studies [5] as well as *in vivo* MRI studies [6], [7]. Degradation of WM microstructure can occur secondary to gray matter (GM) pathology via an accumulation of aggregated hyperphosphorylated tau protein and the deposition of Aβ [8]. There is also evidence for direct WM damage occurring as a result of oligodendrocyte death and reactive gliosis [9]. Another concept of AD pathology is the retrogenesis theory which posits that WM degeneration follows a pattern that is the reverse of myelogenesis with late-myelinating pathways being first affected by AD, and early-myelinating pathways affected later in the disease [10].

The advent of diffusion tensor imaging (DTI) has provided new opportunities to study WM tracts *in vivo* [11]–[13]. DTI exploits the fact that water diffuses faster along the main axis (λ_1_) of fibers compared with diffusion perpendicular to fibers (λ_2_, λ_3_). These properties of diffusion are elaborated through mathematical tensors which enable WM tracts to be analyzed via four main indices of diffusion - fractional anisotropy (FA), mean diffusion (MD), axial diffusion (DA) and radial diffusion (DR) [12], [14]. To date, most studies have concentrated on the role of FA in AD and MCI [15]–[18]. FA values decrease as WM integrity is affected by either normal aging or neurodegenerative processes, but a range of factors influence this decrease, including myelination, axon density, axon diameter and intravoxel coherence of fiber orientation [19], [20]. There is an increasing awareness of the limitations of individual indices, and the need to investigate how multiple indices change over the course of neurodegenerative diseases. The study of DA, DR and MD, in addition to FA, offers a potential new field for exploring the temporal and spatial aspects of the onset and progression of AD. DA refers to diffusion along the direction of the highest eigenvalue (λ1), while DR is calculated as the average diffusion along the two radii (λ2 and λ3) orthogonal to λ1. Animal studies [21] and subsequent human studies [22] have shown that damage to myelin leads to increases in radial diffusion. Increases in DR are thought to be a possible indicator of primary WM damage in AD and also a reflection of demyelination that occurs in normal aging [23], [24]. For DA, both human and animal studies indicate that damage to WM can lead to an initial decrease in DA followed by subsequent increases in DA as axonal fragments are cleared by microglia and water molecules can diffuse longitudinally once again [25], [26]. DA results have been equivocal with both regional increases [6], [7] and decreases [27] reported in AD and MCI. Mean diffusivity (MD) reflects the overall magnitude of water diffusion and is calculated as the mean of all three eigenvalues [12].

DTI has also been used to show that some of the damage to the parahippocampal region in AD may represent a unique WM pathology as decreases in FA and increases in MD remain in this region even after controlling for hippocampal volume loss [28]. In relation to tau pathology, DR has been found to be raised in MCI patients with elevated levels of total tau in cerebrospinal fluid, whereas DR remains unchanged from controls in MCI patients with normal tau values [29].

The goal of the current study was to assess changes in multiple indices of diffusion that occur in amnestic and non-amnestic MCI (MCIa and MCIna respectively), as well as in AD, using an unbiased and hypothesis free whole-brain approach. This was achieved by the method of tract-based spatial statistics (TBSS) [30], which obviates the need for *a priori* selection of regions of interest. To our knowledge no study has yet characterized different diffusion profiles for MCIa and MCIna groups using multiple indices of diffusion. We also assessed overlapping indices as previous studies have suggested that identifying differences in MD, DR and DA in areas where FA decreases may be useful for pinpointing specific types of WM damage in AD [31]–[33]. The study of regional overlaps among WM indices (denoted as conjunctions in this paper) are useful because two identical reductions in FA may be interpreted in different ways depending on which eigenvalues are altered. For example, FA may be reduced via an increase in DR and MD, while DA remains unchanged [6], [34]. FA might also be reduced via a decrease in DA and an increase DR, with no change in MD [6], [34]. Therefore we assessed three specific conjunctions in an effort to extract extra information about WM damage.

Firstly, zones of lower FA together with higher MD were assessed as this conjunction has previously been suggested to indicate reduced microstructural integrity that is associated with macroscopic tissue loss and increased brain water content [31], [35], [36]. MD is particularly sensitive to extracellular volume and the conjunction of lower FA and higher MD may indicate areas where there is a loss of structural barriers to the diffusion of water [37]. Previous studies have reported significant correlations between MD and FA in macroscopic lesions in multiple sclerosis and it has been proposed that tissue loss alone would cause an increase in MD and a decrease in FA [36], [38], [39].

Conversely, areas of lower FA with no increase in MD are thought to highlight microstructural changes to WM fibers that are independent of gross tissue loss [31], [35], [36], [40]. It has been suggested that this situation may arise from subtle differences in DA and DR which reflect minor fiber damage without gross tissue loss [33]. This conjunction has also been associated with the occurrence of gliosis [36], [38].

Lastly, decreased FA together with increased DR was assessed as this conjunction has previously been linked with loss of myelin in human studies [7], [31] and also in animal models of experimentally induced myelin loss [42]–[44]. This conjunction has also been observed in multiple sclerosis where there is acute demyelination [45].

Our hypothesis was that areas of significant damage in MCI and AD would overlap with known networks such as the default mode network which are compromised in AD [46], [47]. We also predicted that there would be subtle differences between MCIna and MCIa which would be highlighted by indices of absolute diffusion (i.e. DR, DA and MD). Recent work has suggested that FA might not be as sensitive as absolute indices of diffusion in locating subtle WM damage [6], [7]. Furthermore, we predicted that a comparison of MCIa versus AD, and MCIna versus AD contrasts, would highlight WM regions that are progressively affected as AD becomes more severe. As the progression of AD follows a predictable pattern, with the earliest changes starting in poorly myelinated areas of the temporal lobe and spreading to the parietal lobe [48]–[52], we expected that both the temporal and parietal lobes would be shown to be compromised in a comparison of MCIna against AD. Only in later stages are more extensive cortical areas compromised, including the densely myelinated primary fields of the neocortex [48]–[52]. Therefore, we expected to see more widespread areas of the neocortex compromised in a comparison of MCIa against AD, which highlights the later stages of the disease.

## Materials and Methods

### Ethics Statement

The study was approved by the St. James' Hospital and Adelaide & Meath Hospital incorporating the National Children's Hospital Research Ethics Committee and was in accordance with the Declaration of Helsinki. All participants provided informed written consent.

### Participants

Scans were obtained from four groups of participants: 40 healthy older people, 19 MCIna, 14 MCIa, 9 AD. The total number of participants was 82. MCI patients were diagnosed using criteria for both amnestic and non-amnestic sub-groups [53]. Neuropsychological assessment consisted of the Mini Mental State Examination (MMSE) [54] and the Consortium to Establish a Registry for Alzheimer's Disease (CERAD) neuropsychological battery [55]. For the diagnosis of MCI, the following must be present:

(1) objective impairment on any neuropsychological test from the CERAD battery based on a cut-off of -1.5 SD below published normative data corrected for age and education of the subject;
(2) cognitive impairment corroborated by a close family member;
(3) essentially normal activities of daily living;
(4) must not meet criteria for dementia as defined below.

MCI individuals with objective memory impairment were diagnosed as having MCIa and those with non-memory impairment were diagnosed as having MCIna.

Diagnostic criteria of AD were that of the National Institute of Neurological Disorders and Stroke–Alzheimer Disease and Related Disorders (NINCDS–ADRDA) working group [56]. AD, MCIna and MCIa participants were recruited at the Adelaide and Meath Hospital incorporating the National Children's Hospital (AMNCH), Dublin, Ireland. Healthy participants were recruited among relatives of patients and also through advertisements in the local community.

Participants were excluded if they had cortical infarction, excessive subcortical vascular disease, space-occupying lesions, depression, and any other psychiatric or neurological disease. Participants were also excluded on magnetic resonance imaging criteria such as pacemaker implant, recent metallic implants, and claustrophobia.

### Imaging Methods

Magnetic resonance imaging (MRI) was conducted with a Philips Achieva 3.0 Tesla MR system (Best, The Netherlands). A parallel SENSitivity Encoding (SENSE) approach [57] was used. The high resolution 3D T1-weighted structural images were achieved with the following pulse sequence: TR = 8.4 ms; TE = 3.9 ms; flip angle  = 8°; number of axial slices  = 180; slice thickness  = 0.9 mm; acquisition voxel size  = 0.9×0.9×1.8 mm^3^; rec voxel size  = 0.9×0.9×0.9 mm^3^; field of view (FOV)  = 230 mm×230 mm ×230 mm; acquisition matrix  = 256×256; SENSE reduction factor  = 2.3; total acquisition time  = 5 min 44 sec.

DTI was acquired using an echo planar imaging (EPI) sequence with the following pulse sequence: TR = 12396 ms; TE = 52 ms; acquisition voxel size  = 2×2×2 mm^3^; rec voxel size  = 1.75×1.75×2 mm isotropic, 60 axial adjacent slices; slice thickness  = 2 mm (no gap); FOV  = 224 mm×224 mm ×120 mm; acquisition matrix  = 112×112; SENSE reduction factor  = 2, combined with a half-scan acquisition; 1 image without diffusion weighting and 15 diffusion-encoding gradients applied in 15 noncollinear directions; b-value  = 800 s/mm^2^; both the b0 and the 15 diffusion weighted images were averaged twice; bandwidth  = 2971 Hz/pixel; total acquisition time  = 7 min 34 sec.

A T2-weighted fluid attenuation inversion recovery (FLAIR) sequence was also acquired to ensure that vascular pathology was not significant. All images were rated using the Fazeka scale [58]. The mean and SD for all participants was 1.33, SD: 0.71; while specific subgroups were as follows; Controls: 1.18, SD 0.51; MCIa: 1.08, SD 0.28; MCIna: 1.37, SD 0.83; AD: 2.22, SD: 0.97.

### DTI Processing

DTI analysis was performed using TBSS [59]. Images were skull stripped with the Brain Extraction Tool (BET) from the FSL library [60]. Raw DTI images were first corrected for motion and eddy current effects. The diffusion tensor was then calculated with the DTIFIT program for whole brain volumes and the resulting FA maps, together with the DA (λ1) and DR ((λ2+ λ3)/2) and MD ((λ1+ λ2+ λ3)/3) maps, were used in subsequent TBSS analysis.

TBSS performs a non-linear registration which aligns each FA image to every other one and calculates the amount of warping needed for the images to be aligned. The most representative image is determined as the one needing the least warping for all other images to align to it. The FSL library also provides a 1 mm isotropic FA target image (FMRIB58_FA) in standard space which sometimes used instead of the most representative image from the study cohort. This can be problematic as the target image is based on a young healthy brain. Using the method of “all subject to all subject” registration is more computationally intensively, but highly desirable when dealing with populations other than young healthy controls.

After this registration step, warped versions of each subject's FA image were generated which were then averaged and a white matter “skeleton” is created suppressing all non-maximum FA values in each voxel's local-perpendicular direction and subsequently comparing all remaining non-zero voxels with their nearest neighbours, thus searching for the centre of fibre bundles. The skeleton was then thresholded at an FA value of 0.2 which limits the effects of poor alignment across subjects and reduces the likelihood of inclusion of GM and CSF voxels in the skeleton. The skeleton that is now created contains WM tracts that are common to all subjects. A “distance map” is then created which is used to project each FA image onto the mean FA skeleton that is common to all subjects [59]. The same non-linear transformations derived for the FA maps were applied to the DA, DR and MD maps.

For statistical analysis, the images were analyzed using the “randomise” tool from FSL using a standard GLM design which controlled for the effect of age and gender. Randomise computes permutation tests on the assumption that the null hypothesis implies complete exchangeability of the observations [61]. Using this setup voxelwise differences between groups were then assessed, setting the number of permutations at 5000 permutations. Significance was tested at p<0.05 levels, corrected for multiple comparisons family wise error comparison (FWE). Cluster-like structures were enhanced using a recently proposed method called threshold-free cluster enhancement [62]. This method obviates the need to specify an arbitrary threshold in order to quantify areas of significant difference between contrasts. Areas of significant difference were visualized using the 3DSlicer program (http://www.slicer.org/).

## Results

### Demographic and Cognitive Characteristics

Healthy elders, MCIna, MCIa and AD patients did not differ in terms of age or education. MMSE was significantly lowered in AD subjects compared with the other three groups. MCIna subjects contained significantly more females than males, but for all other groups the gender distribution was not significantly different (Table 1).

**Table 1:** Demographic and Cognitive Characteristics of the Sample Groups.

		Chi-Sq									
Groups	n	p-value	Age			Education			MMSE		
Control	40	0.2	66	±	8	13	±	5	29.27	±	1.11
M	16		68	±	8	14	±	7	29.25	±	1.44
F	24		64	±	7	12	±	2	29.29	±	0.86
MCIna	19	0.039	68	±	6	12	±	2	28.26	±	2.86
M	5		71	±	7	12	±	2	27.80	±	2.39
F	14		67	±	6	12	±	2	28.43	±	3.08
MCIa	14	1.000	68	±	8	13	±	4	27.93	±	3.69
M	7		71	±	8	12	±	4	27.71	±	3.59
F	7		65	±	8	15	±	4	28.14	±	4.06
AD	9	0.739	70	±	9	12	±	3	**20.89**	±	3.86*
M	4		70	±	10	10	±	2	19.75	±	1.89
F	5		70	±	9	14	±	2	21.80	±	4.97

Values are mean ± standard deviation. Abbreviations: MCIna, non-amnestic Mild Cognitive Impairment; MCIa, amnestic Mild Cognitive Impairment; AD, Alzheimer's Disease; MMSE, Mini-Mental State Examination. *Post-hoc analysis with Bonferroni correction: p<0.05 in AD versus CON; AD versus MCIna; AD versus MCIa.

### Early detection of diffusional changes in MCI

**Figure 1:**
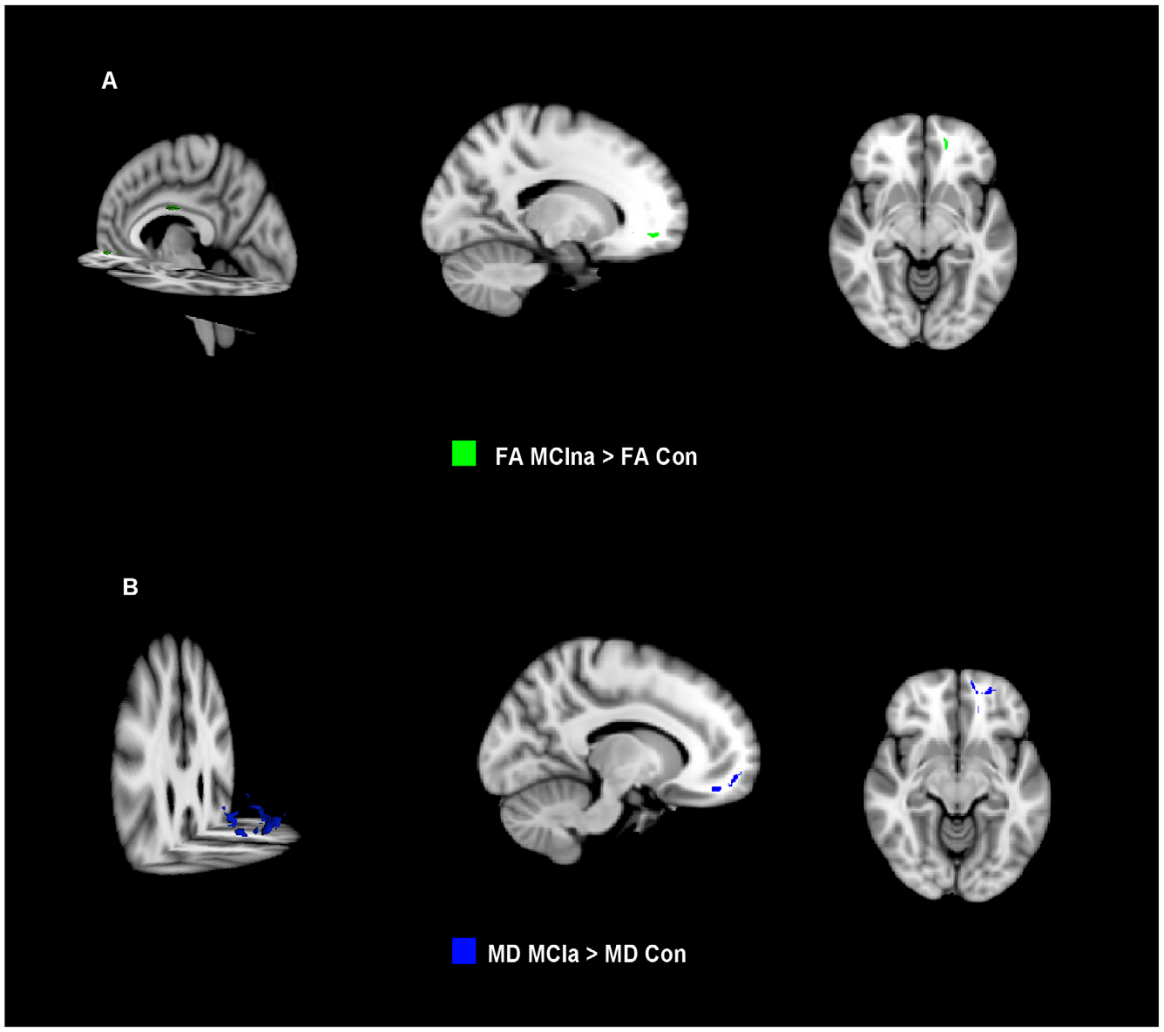
Diffusional changes in MCI subjects for FA and MD. (a) Significantly higher FA in MCIna relative to controls is shown in green. (b) Significantly higher MD in MCIa relative to controls is shown in red.

**Figure 2:**
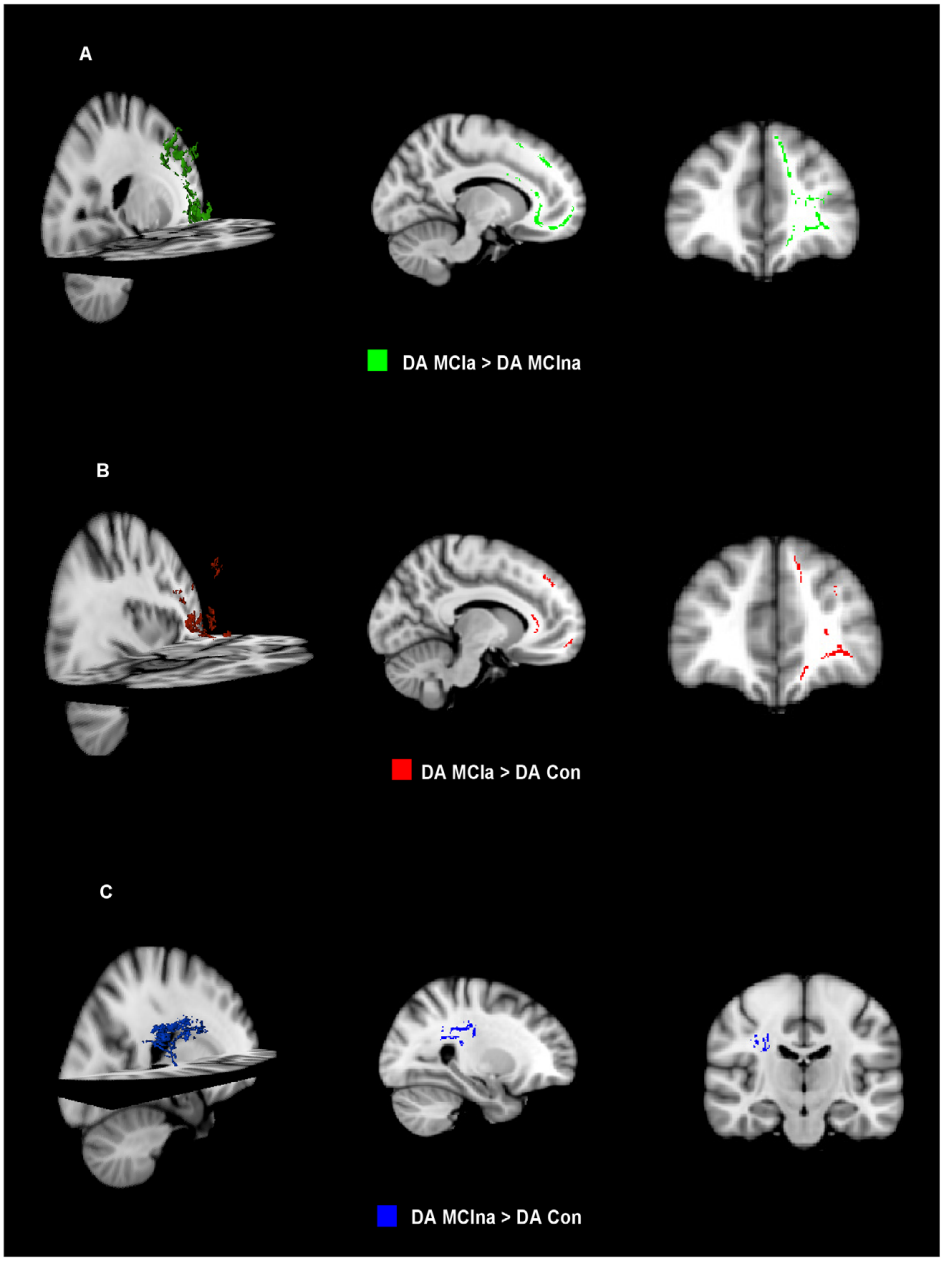
DA changes in MCIna and MCIa subjects. (a) Significantly higher DA in MCIa relative to MCIna. These significant changes are found in the left hemisphere in frontal regions. (b) Significantly higher DA in MCIa relative to control (red). These significant changes are also found in the left hemisphere in frontal regions. (c) Significantly higher DA in MCIna relative to control (blue). These significant changes are found in the right hemisphere in the parietal lobe.

Small areas of significantly higher FA were found in MCIna relative to control in the midbody of the corpus callosum (Figure 1). DA was found to be significantly higher in MCIna subjects relative to control subjects in the right posterior cingulum/precuneus (Figure 2).

For differences between control and MCIa, significantly higher DA was only detected in left frontal regions, including the forceps minor and parts of the left prefrontal cortex (Figure 2). The MD index detected significantly higher values in MCIa relative to control in the left forceps minor (Figure 1). DA was also found to be significantly higher in MCIa relative to MCIna in parts of the left prefrontal cortex (Figure 2). For a summary of significant differences between all contrasts see Table 2.

**Table 2:** Summary of Results Obtained with TBSS with all 4 indices.

	CON:MCIna	CON:MCIa	CON:AD	MCIa:AD	MCIna:AD	MCIa:MCIna
FA	268	x	72,029	57,871	40,727	x
DA	1,182	1,123	36,236	9,356	24,998	3,756
DR	x	x	81,157	49,119	60,402	x
MD	x	290	76,036	35,301	53,980	x

The total volume of significant voxels for each contrast is given in mm^3^. Results are calculated on the total volume of voxels remaining in each contrast at a significance level of p<0.05, corrected for multiple comparisons with family wise error comparison. x = no significant result. CON; Control, MCIna; non-amnestic MCI; MCIa; amnestic MCI. Each contrast is denoted by “:”; i.e. CON:MCIna refers to the control versus MCIna contrast.

### WM changes between Control and Alzheimer's Disease

**Figure 3:**
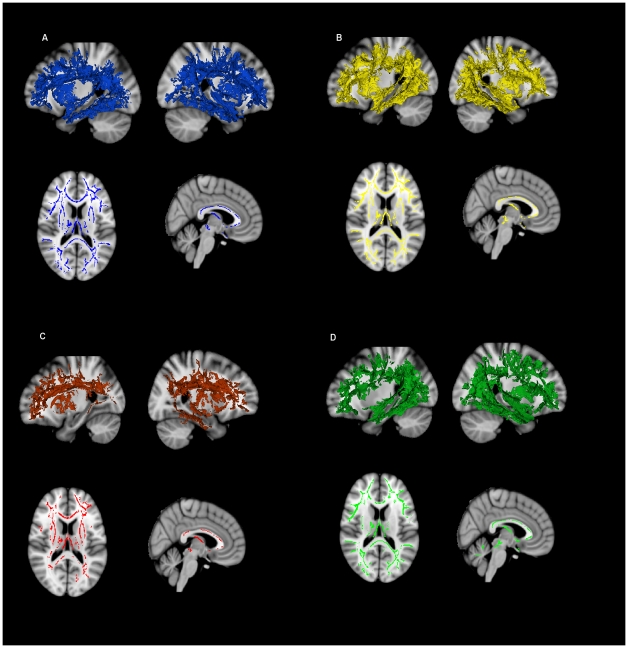
Differences between AD and control subjects for all indices. (a) MD diffusivity, (b) DR diffusivity, (c) DA diffusivity, (d) FA diffusivity. For each index a three dimensional model of the significant changes are shown for the left and right hemispheres in the upper panels. In the lower panels, an axial slice and a sagittal slice show specific changes located at the level of the slice.

Significant voxelwise differences between control and AD groups were found in WM tracts for all indices. Diffusion differences between control and AD groups, overlapped widely in all indices, and included the temporal lobe and parahippocampal gyrus, parietal lobe, prefrontal cortex, as well as the uncinate fasciculus, the anterior and posterior corpus callosum, the anterior and posterior cingulum and the fornix (Figure 3).

**Figure 4:**
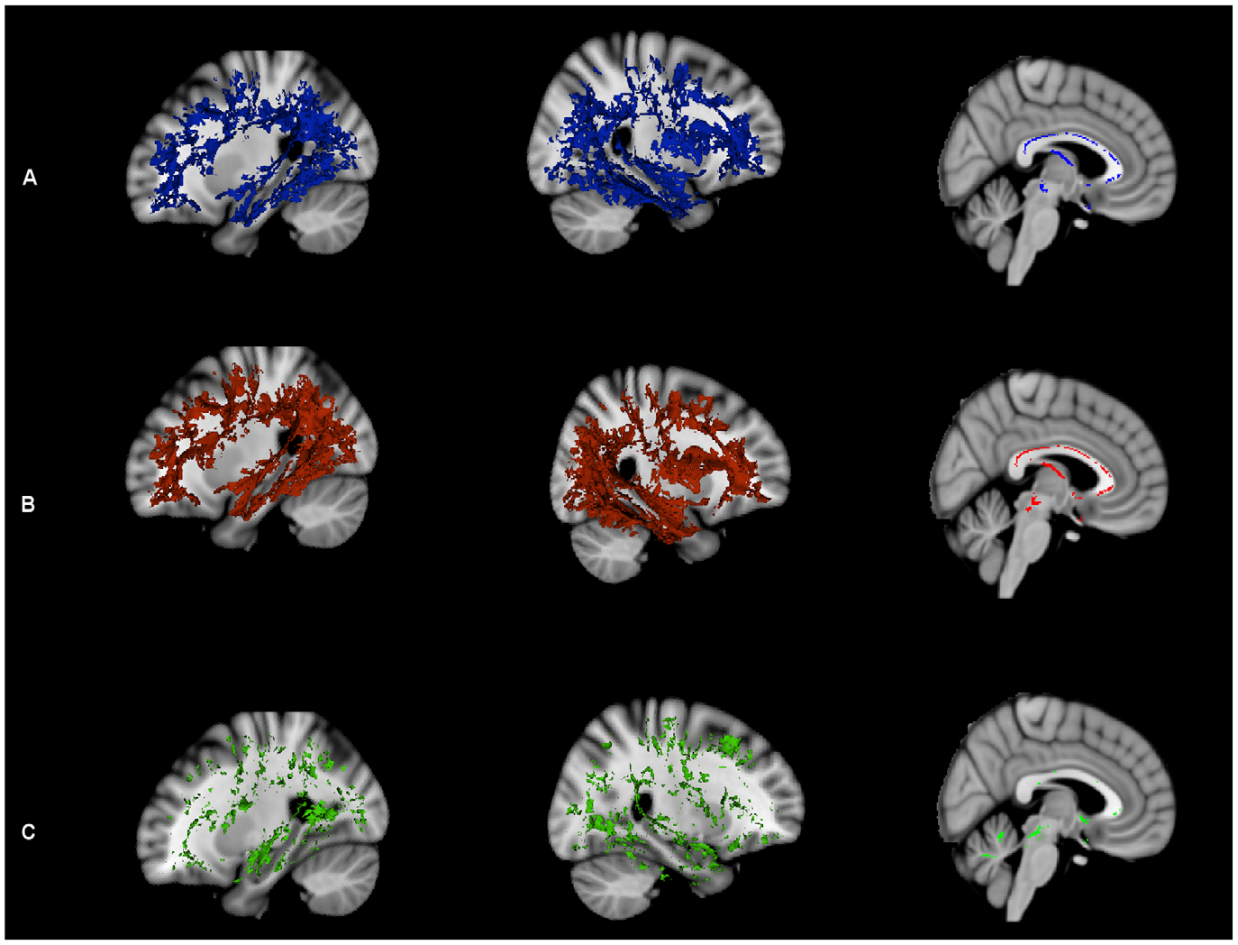
Differences between AD and control subjects for specific overlapping indices. (a) Gross tissue loss is indicated by areas where FA decreases overlap with MD increases. (b) Myelin damage is indicated by areas where FA decreases overlap with DR increases. (c) Microstructural damage in the absence of gross tissue loss is assessed by areas where FA decreases but MD remains unchanged. The left panels show three-dimensional models of the left hemisphere. The middle panels show three-dimensional models of the right hemisphere. The right panels show areas of changes restricted to a single sagittal slice to highlight the situation for the corpus callosum. See text for further details regarding overlapping indices.

Significant voxelwise differences between control and AD were also found for the following combinations of indices: (i) lower FA together with higher MD, indicative of gross tissue loss (denoted by FA ↓, MD ↑); (ii) lower FA together with higher DR, indicative of myelin loss (denoted by FA ↓, DR ↑); (iii) lower FA where MD remains unchanged, indicative of microstructural changes that occur independent of gross tissue loss, (denoted by FA ↓, MD –) (Figure 4). For a detailed interpretation of these overlaps see Introduction and Discussion. The areas implicated by (i) FA ↓, MD ↑, and (ii) FA ↓, DR ↑, were comparable to the widespread areas of the temporal, parietal and frontal lobes highlighted by individual indices. Again, the core regions the parahippocampal tract, the posterior cingulum/precuneus, corpus callosum, fornix and prefrontal cortex were shown to be affected by these two overlaps. In contrast, the FA ↓, MD –, analysis, identified a smaller area. The corpus callosum and fornix were not affected in the FA ↓, MD – results, while there was a relative sparing of the hippocampal tract. However, dispersed regions through the temporal lobe, posterior parietal lobe, and prefrontal cortex were still affected.

**Figure 5:**
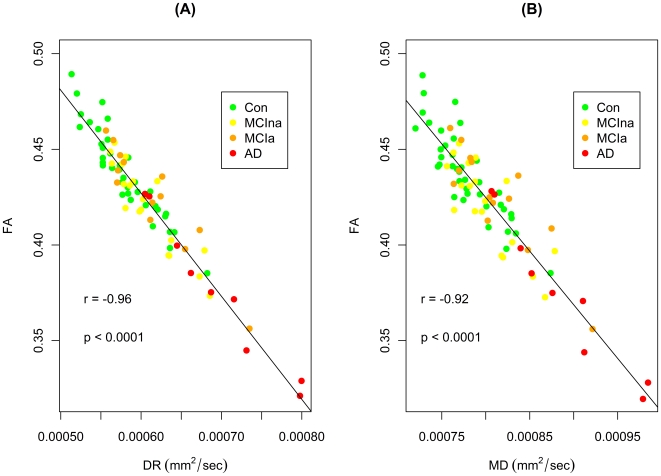
Analysis of correlation between diffusion indices. (a) Correlation between FA and DR within a mask of the regions implicated in Figure 4 (b) where FA was significantly lower and DR was significantly higher in AD subjects relative to controls. (b) Correlation between FA and MD within a mask of the regions implicated in Figure 4 (a) where FA was significantly lower and MD was significantly higher in AD subjects relative to controls. The r value denotes the correlation as calculated by Pearson's product-moment correlation using data from all subjects. Correlations are significant as indicated by the p values.

In order to further characterize the relationship between FA and DR, and also between FA and MD, the correlations between these indices were also assessed. A mask was created for the areas implicated in Figure 4 (b) where there was lower FA and higher DR in AD subjects relative to controls, and a strong inverse correlation between FA and DR was found within this mask (p<0.0001, r = −0.96, Figure 5 (a)). For the areas implicated in Figure 4 (a) where there was lower FA and higher MD in AD subjects relative to controls, a mask was also made for these regions and a similarly strong inverse correlation between FA and MD was found within this mask (p<0.0001, r = −0.92, Fig. 5 (b)).

### WM changes between MCIa versus AD, and, MCIna versus AD

**Figure 6:**
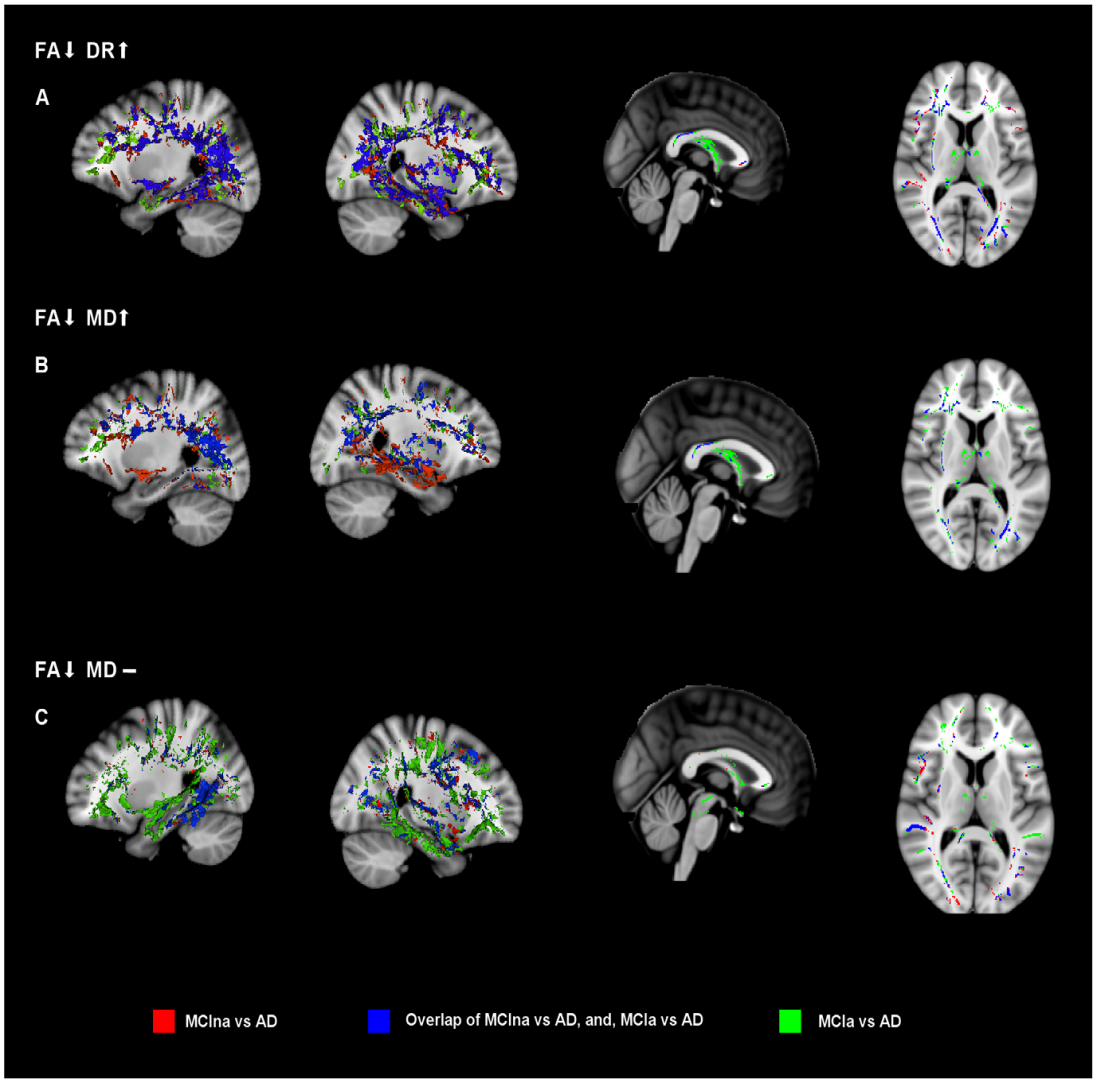
Analysis of disease progression with MCIna versus AD, and, MCIa versus AD. MCIna versus AD is shown in red, MCIa versus AD is shown in green, and areas where these contrasts overlap is shown in blue. (a) FA decreases together with DR increases. (b) FA decreases together with MD increases. (c) FA decreases where MD remains unchanged. See text main text for anatomical description and explanation of the three combinations of indices.

The three combinations of indices listed above were assessed for the MCIa versus AD, and also for MCIna versus AD contrasts (Figure 6). Results indicated that diffusional changes due to myelin damage (i.e. FA ↓, DR ↑ changes, Figure 6 (a)) were prominent in temporal and parietal lobes, particularly in the posterior cingulum and hippocampal tract, for both MCIna versus AD, and MCIa versus AD contrasts. The fornix was shown to be affected only in the MCIa versus AD contrast, indicating this structure is affected by myelin damage in later stages of AD.

The FA ↓ MD ↑ overlap (Figure 6 (b)), which indicates diffusion changes due to gross tissue loss, was not as widespread, but the posterior cingulum/precuneus and parts of the anterior cingulum and frontal lobe were significantly affected in both contrasts. The fornix again showed changes only for the MCIa versus AD contrast indicating that diffusional changes in this structure occur in later stages of AD and may be driven by gross tissue loss.

FA decreases where MD remains unchanged (FA ↓, MD – ), indicative of microstructural changes that are independent of gross tissue loss, are shown in Figure 6 (c) for both contrasts. Changes in the MCIa versus AD were more prominent than changes in MCIna versus AD, suggesting that microstructural changes that are independent of gross tissue loss occur to a large extent in later stages of neurodegeneration. The fornix was relatively spared for both contrasts, while MCIa versus AD changes occurred in hippocampal tract, as well as in dispersed areas of the prefrontal cortex posterior cingulum and anterior cingulum. MCIna versus AD changes were largely confined to the left hippocampal tract.

## Discussion

Our findings indicate that early neurodegenerative changes occurring in MCIna and MCIa can be detected with multiple indices of diffusion. Our findings also highlight small but distinct differences in DA between MCIna and MCIa subjects. Significantly higher DA in MCIna subjects relative to controls was found in the right posterior cingulum. Two small regions of higher FA in MCIna subjects relative to control were found in the mid-body of the corpus callosum and ventral prefrontal cortex of the left hemisphere. For MCIa subjects, significant changes were found in the left prefrontal cortex with DA and to a smaller extent with MD.

The posterior cingulum/precuneus is an important hub which sustains information transfer between the parahippocampal gyrus and the prefrontal cortex [63] and our findings agree with a number of studies which have proposed that damage to the posterior cingulum/precuneus leads to dysfunction of a network that is responsible for sustaining memory function [6], [28], [47], [63]. The precuneus is connected to the temporal lobe via the retrosplenial cortex, and the hippocampus may not be able to communicate with the neocortex if it is sufficiently damaged [28], [63]. PET studies have also highlighted reduced metabolism in the posterior cingulum and the prefrontal cortex in both MCI and AD subjects [64], [65]. The atypical increase in FA in two isolated regions of MCIna subjects may be related to the presence of crossing fibers, as one previous study noted that in zones of crossing fibers the sparing of one tract relative to the other can produce increases in FA [66].

Interestingly, DA was also significantly higher in MCIa relative to MCIna in the left dorsal and ventral prefrontal cortex, including the forceps minor and uncinate fasciculus. This may indicate increased neurodegeneration in MCIa subjects relative to MCIna, which would concur with the fact that memory impairments are part of the MCIa condition while MCIna subject lack such impairments. Furthermore, the prefrontal cortex has frequently been associated with the retrieval of autobiographical memories [67], [68]. Although no other work using TBSS has looked at both MCIna and MCIa, our findings concur with previous work that has found DA increases in MCI [69] and AD [6], [28]. Bosch et al. found DA increases in AD, but not in MCI [7]. However, another study has shown DA *decreases* in both MCI and AD [27].

The complexity of DA responses may reflect the fact that WM damage has been shown to lead to an initial decrease in DA followed by subsequent increases as axonal fragments are cleared by microglia and water molecules can diffuse longitudinally again [25], [26]. An alternative explanation may relate to the presence of crossing fibers. Where two crossing fibre tracts exist, it may be difficult to dissect out the axial diffusion of one of the two fibre tracts. Thus, increases in DA may reflect a degeneration of one of the two fibre populations in regions of crossing fibers [66].

Despite these complexities, a common finding is that early WM damage appears to be localized to the posterior cingulum and prefrontal cortex, particularly the uncinate fasciculus, at an early stage in neurodegeneration. It is of note that the forceps minor, uncinate fasciculus, and posterior cingulum all myelinate at a late ontogenetic stage, which has been suggested to leave them vulnerable to attack in the early stages of neurodegeneration [10], [70]. The uncinate fasciculus connects the hippocampus to the subgenual cortex, and damage to this tract has been shown in MCI and AD [64], [71], [72]. The posterior cingulum and prefrontal cortex are also components of both the episodic memory network and the default mode network [46], [67], a fact which also suggests that damage to these regions is likely to lead to memory impairments [73].

To the best of our knowledge, no previous studies have investigated differences between MCIa and MCIna using multiple indices. A study using only FA found differences between control and MCIa subjects in frontal, temporal, parietal and occipital lobes, while differences between control and MCIna subjects were more diffuse with the temporal lobe being relatively spared [74]. However, the subjects in that study were approximately ten years older than those under consideration here (i.e. ∼77 years compared with ∼68 years). At younger ages it is more difficult to detect subtle changes in WM, and the use of multiple indices, as outlined here, facilitates the detection of early damage to WM tracts. Our results also add support to recent work suggesting that changes in absolute diffusion (DA, DR, MD) may be more sensitive than FA at detecting WM changes in neurodegeneration [6], [7]. An over reliance on FA is therefore a possible contributing factor in the equivocal nature of DTI results in MCI subjects.

For AD subjects, significant changes were present in all indices and regions affected including the temporal, parietal and occipital lobes, as well as the corpus callosum, the anterior and posterior cingulum, the fornix, and the dorsal and ventral prefrontal cortex. FA was significantly decreased in AD, while the three other indices increased significantly. There was significant overlap between all indices, with MD and DR overlapping most closely. While DA increases were found in the areas noted above, they were not as widespread as the other indices, and DA also showed a relative sparing of the occipital lobe.

Previous work has found significant FA decreases in AD using TBSS [6], [7], [28], [75], while one study failed to find FA changes in AD [6]. The few studies which have looked at multiple indices in AD have reported similar changes as reported here for DR, MD and DA [6], [7], [28].

Taken together our results suggest that when AD is fully manifest WM damage is widespread and extends beyond the central hubs of the posterior cingulum and uncinate fasciculus that were noted in MCI analysis. The fornix was also significantly affected in all indices, a finding which concurs with previous work [6], [7], [65], [76]. As the fornix connects the mesial temporal lobe and diencephalon and is an integral part of the circuit of Papez, it has been noted as having a key role in the pathogenesis of AD [6], [65].

Further analysis assessed areas where significant changes in FA and DR overlap (termed FA ↓, DR ↑ from here on), which is thought to highlight specific myelin damage [21], [31], [41], [42], [44]. Areas affected by this overlap were found to be widespread, including the parahippocampal tract, parietal lobe, posterior cingulum, corpus callosum, fornix and frontal lobe. Thus significant damage of myelin is evident in AD which was not found at the MCI stage, and this is consistent with reduced myelin staining in AD [77]. The overlap of significant changes in FA and MD (termed FA ↓, MD ↑ from here on), which is indicative of the accumulation of water and gross tissue loss [31], [35], coincided to a large extent with areas where myelin damage was highlighted, and included the parahippocampal tract, the posterior cingulum, anterior cingulum, fornix, corpus callosum and prefrontal cortex. It is possible that Wallerian degeneration secondary to distal cortical atrophy may be driving WM changes in some of these regions, particularly the fornix which is the major efferent tract of the hippocampus [78]. Previous work which combined DTI with hippocampal volume suggests that WM damage in AD may result from both Wallerian degeneration as well as direct damage to myelin which is independent of secondary degeneration [28]. While the current study has not combined DTI with volumetric analysis, the presence of diffusion changes independent of gross tissue loss, as evidenced by lower FA in areas where MD is unchanged might lend some support to the idea that Wallerian degeneration alone is unlikely to account for all WM damage. These changes were more limited than those for FA ↓, MD ↑ and FA ↓, DR ↑, but included parts of the left inferior longitudinal fasciculus, left parahippocampal tract, and dispersed areas of the parietal and frontal lobes.

The relationship between different indices was examined in further detail by means of correlation analyses. Results revealed a strong inverse correlation between FA and DR within a mask created for the conjunction of lower FA and higher DR in the control versus AD contrast. A similarly strong inverse correlation was also found between FA and MD within a mask of the conjunction between lower FA and higher MD. Previous work has shown significant inverse correlations between FA and MD in multiple sclerosis (MS) lesions [38], [39]. However, we should bear in mind that our analysis of regional overlaps also revealed dispersed areas where there is reduced FA together with unchanged MD in AD patients. Interestingly, studies in MS have suggested that increased gliosis could lead to decreases in both FA and MD, which would reduce the inverse correlation between these two indices [39]. Such a change in the slope of this correlation could be assessed in longitudinal AD studies which may provide an indication of changing levels of gliosis as neurodegeneration progresses. Previous studies have reported a high degree of gliosis in both normal aging and dementia [8], [79].

Two further contrasts, MCIna versus AD, and, MCIa versus AD, were assessed using (1) FA ↓, DR ↑, (2) FA ↓, MD ↑ and (3) FA ↓, MD –, measures. Overlaying these two contrasts allows us to examine areas where disease progresses into as neurodegeneration becomes more severe in the MCIa versus AD contrast.

For measures of diffusional change associated with gross tissue loss (FA ↓, MD ↑) and myelin damage (FA ↓, DR ↑), the core regions of the hippocampal tract, posterior and anterior cingulum, and prefrontal cortex were affected in both contrasts. However, the fornix was only implicated in the MCIa versus AD contrast suggesting that in later stages of AD Wallerian degeneration may have an impact on this structure.

The situation for microstructural damage in the absence of gross tissue loss (FA ↓, MD –) was substantially different. These diffusional changes appear to expand from a core area of the hippocampal tract in the MCIna versus AD contrast, to a wider network including much of the temporal lobe, as well as the posterior and anterior cingulum in the MCIa versus AD contrast. This result indicates that primary WM damage appears to expand greatly as neurodegeneration progresses.

The classical view of WM changes in AD suggests that anterograde Wallerian degeneration is the primary driving force with regions close to cortical areas being most affected [15], [80], [81]. An alternative view suggests that direct damage to WM can occur in both aging and AD [4], [82]. A large body of evidence suggests that damage to myelin may be one of the earliest events in AD, and Bartzokis has proposed that AD may begin as a disease of demyelination, with amyloid and tau accumulation arising as by-products from homeostatic repair mechanisms that are activated by this demyelination process [82]. While our results are not capable of directly distinguishing between these two theories, our analyses suggest that direct damage to myelin, as well as Wallerian degeneration, may both play a role in AD. Late-myelinating pathways have been shown to be particularly vulnerable to direct WM damage [10] and results for the forceps minor, uncinate fasciculus and posterior cingulum in the current study agree with this. However, as we have seen, the WM damage in the fornix is more likely to be driven by Wallerian degeneration as indicated by the overlap of FA decreases and MD increases in this tract.

Our analysis of MCI subjects also points to WM damage in the late-myelinating pathways of the uncinate fasciculus, posterior cingulum and forceps minor [4], [10], [82]. This is concordant with the retrogenesis theory which posits that tracts that are late to myelinate in ontogenetic development are among the earliest to be affected in AD [4], [82].

In summary, our approach identifies different diffusion profiles for MCIna, MCIa and AD groups, while the use of multiple indices in combination with an analysis of MCIna vs AD and MCIa vs AD contrasts indicates that as disease progresses direct damage of myelin appears to increase markedly in the temporal lobe, parietal and frontal lobes. Our analysis also indicates that the fornix is significantly affected in more serious disease states and that Wallerian degeneration may be responsible for this.

Connections between the temporal and parietal lobes may breakdown due to synapse failure in the hippocampal tract, which in turn leads to neurodegeneration spreading through the parietal lobe, which compromises communication between the medial temporal lobe and the neocortex [1]. Our results are concordant with studies which have shown that the fornix, which is the main efferent pathway of the hippocampus, is significantly compromised in AD [6], [7], [28].

A limitation of the current work is the low number of AD participants included in the study. Bearing this in mind, the primary focus was to understand the potentially transitional MCI state in greater detail. Also, while the TBSS approach strives to avoid the problems of voxel based morphology relating to partial voluming, some of these issues may still remain. Small WM tracts may be contaminated with GM if the tract width is smaller than the original voxel size [59]. Applying a threshold of 0.2 or less to the WM skeleton in the TBSS preprocessing steps is thought to remove the potential occurrence of GM but there is no certainty that all GM contamination is not removed. A further limitation is the fact that we do not have follow-up data and thus do not know which participants progressed to develop AD or other forms of neurodegenerative diseases.

We conclude that as the number of people developing MCI is increasing dramatically on a global scale, it is vitally important to develop an accurate early structural diagnostic marker which will be able to pin-point the onset of AD in this vulnerable and heterogeneous at-risk group. The use of a range of diffusion modalities is likely to provide a fruitful strategy for determining where, how and why the first changes in WM are occurring in the brain.
